# Breast cancer in men: a serie of 45 cases and literature review

**DOI:** 10.11604/pamj.2020.36.183.22574

**Published:** 2020-07-14

**Authors:** Marwa Methamem, Imen Ghadhab, Samir Hidar, Raja Briki

**Affiliations:** 1 Department of Gynecology and Obstetrics, Farhat Hached´s University Hospital, Sousse, Tunisia

**Keywords:** Cancer, breast, men, pathology, imunohistochemistry

## Abstract

Immunohistochemical profiling studies carried out on female breast cancer has been extrapolated to breast cancer in males. Although, we do not know if it really reflects the reality of this pathology in males patients since the studies are often retrospective and studying a limited number of patients. The objectives was to describe particualrities of breast cancer in males and analyze the evolutionary characteristics and study the molecular profile of this rare disease in Tunisian men. It is a retrospective, descriptive and analytic study carried out over a period of 15 years in the departments of gynecology-obstetrics, general surgery, medical carcinology and anatomopathology of the Farhat Hached Teaching Hospital in Sousse, Tunisia. Fourty five patients were included.The most common histological type was invasive ductal carcinoma (95% of our patients). Our series was divided into 3 immunohistochemical groups with a majority group: luminal A (68.2%), followed by luminal B (27.3%) and only one patient had a triple negative type tumor (4.5%).The Overall survival rate (OSR) at 5 and 10 years was 83.2% and 76.8% respectively. Recurrence-free survival (RFS) at 5 and 10 years was 64.5% and 58.6%, respectively. The OSR was influenced significantly by age, clinical and histological size of the tumor, the presence of distant metastases and the occurrence of recurrence. Recurrence-free survival (RFS) was influenced by age, clinical and histological size of the tumor, and infiltration of the dermis. Breast cancer in males has similarities with women's breast cancer. However, it remains diagnosed at a later stage.

## Introduction

In the world, breast cancer is the first malignant tumor in women. However, it is relatively rare in men that it presents less than 1% of cases of male neoplasia and 1 to 2% of all breast cancers [1]. In Tunisia, its frequency is estimated at between 2 and 5% of breast cancers and 1.6% of cancers in men [2]. Breast cancer in men is an unknown pathology to the general public and the discovery of a breast nodule in a man does not cause the same concern as in a woman since the majority of people have trouble realizing that such cancer can occur on a male breast. In women, the frequency of breast cancer has led to many studies to better understand this pathology. On the other hand, in men, and in spite of the numerous studies which refer to this pathology, they are often retrospective studies studying a limited number of patients. Recommendations for the management of male breast cancer are therefore extrapolated from those used for female breast cancer. Men are treated like menopausal women. But, to date, no study based on male population has validated these modalities of treatment in terms of impact on survival. The objectives of this study are based on the description of the epidemiological, clinical, histological and therapeutic characteristics of breast cancer in men, as well as the search for predictive factors of poor prognosis in order to improve the management of these patients.

## Methods

This is a retrospective, descriptive and analytic study of 45 cases of breast cancer in men, based on data from the central cancer registry, collected at the department of anatomy and pathological cytology of the Farhat Hached hospital in Sousse for a period of 15 years (January 2002-December 2016). This is a comprehensive study conducted on 45 patients who were managed during the study period for primary breast cancer, confirmed by a histological examination for whom, the same classification TNM and the same grading histopronostic of Scarff Bloom and Richardson were used. All cases of cancer were treated and followed in the gynecology obstetrics and / or medical oncology and / or radiotherapy and / or dermatology and / or general surgery departments at Farhat Hached Hospital or Sahloul University Hospital of Sousse, Tunisia. Data collection was based on patient medical records, pathology anatomy reports, and outpatient monitoring records. Some missing data was collected by telephone communications.

**Statistical analysis:** the data entry was done with the chi2 test on the SPSS computer program. Statistical analysis was performed using SPSS version 20 software. For the comparison of the variables, it is assumed that the correlation is significant for a probability rate higher than 95% (p < 0.05). The second part includes an univariate analysis of overall survival and survival without recurrence according to different parameters (age, clinical status, location, SBR grade and some histological factors, immunohistochemical subtypes). Overall survival and survival without recurrence were calculated using the Kaplan-Meier method. The comparison of the survival curves according to the prognostic factors was made according to the Log-Rank test. The correlation between clinical and histological tumor size was assessed by calculating the sensitivity, specificity, positive predictive value (PPV) and negative predictive value (NPV) of the clinical estimate of tumor size. For the concordance analysis, we used the kappa test (?).

## Results

During the study period, male breast cancer accounted for 0.8% of breast cancers diagnosed in both sexes in the Sousse region and 0.26% of all male cancers. The incidence of breast cancer in men in the region of Sousse was 1.3 / year, which ranks the penultimate male cancer in terms of incidence. The mean age of the patients was 62.7 ± 15.3 years, and the median age was 63, with extremes of 37 and 92 years. The predominant age group was 50 to 70 years old, accounting for 44.4% of the study population. The average consultation time was 10.8 ± 13.4 months; the median was 5 months, with extremes of 1 month and 5 years. The reasons for consultation were dominated by the discovery of a breast nodule, reported by 77.8% of patients. On the other hand, mastodynia was noted in 20% of patients, ulceration and axillary lymphadenopathy constituted the circumstances of discovery in 11.1% of cases and 8.9% of ccases onsulted for gynecomastia. The clinical characteristics of patients are summarized in Table 1. Echo-mammography was performed in 37 out of 45 patients, demonstrating radiological signs of malignancy in all cases. Mammography revealed nodular opacity in the majority of cases (86.5%), stellar opacity in 13.5% of cases and breast hyperdensity in 5.4% of cases. Cutaneous thickening was noted in 27% of patients and plurifocality in 4.4% of cases. Hypoechoic lesions were the most frequently detected ultrasound image (70.3%). Histological confirmation was obtained by performing a microbiopsy in more than half of the cases (55.6%) and a biopsy in 15.6% of the patients. Five patients were operated immediately (11.1%). It was a radical surgery in four of them, and a lumpectomy in the last one. The extension assessment made in 43 patients (95.5%), was normal in 34 of them, and in favor of metastases in 7 patients (15.6%). According to the Union for International Cancer Control (UICC) classification, the predominant tumor stages were represented by Stage IIIB and Stage IIA, with 26.7% of cases each. The histological and immunohistochemical characteristics are summarized in Table 2. Thirty eight patients were treated surgically (84.4%). It was primary surgery for 30 of them, and after neoadjuvant chemotherapy for eight patients. Surgical treatment was radical in 84.2% of patients and conservative in 13.2% of cases. A final mastectomy was performed in a single patient (2.6%). Axillary dissection was performed for 37 patients, in 97.4% of operated cases. For the sentinel lymph node technique, it was performed in three patients (7.9%), followed by axillary dissection in all cases. The ganglionic invasion rate and 67.5%, the average number of ganglia invaded was 6.1 ± 8.9 nodes with extremes of 0 and 30 ganglia invaded. Thirty-four patients received chemotherapy (75.5%), adjuvant in 57.8% of cases. Of the nine patients receiving neoadjuvant chemotherapy (20%), five received adjuvant chemotherapy as well. Locoregional radiotherapy was administered in 55.6% of cases. Twenty-three patients received hormone therapy (51.1% of cases). Twenty-seven of these were tamoxifen and one patient only aromatase inhibitors (Arimidex). The occurrence of recurrence marked the post-treatment evolution of 10 patients, in 26.3% of the operated patients: one case of locoregional recurrence and 9 cases of distant metastases and whose pulmonary involvement was predominant (55.5%). This recurrence occurred after an average of 43 ± 22.6 months of surgical treatment, a median of 48 months, and extremes of 4 months and 7½ years. Sixty percent of patients recurred two years after surgery. After an average follow-up of 44.7 months, a median follow-up of 30 months and extremes of 3 months and 15 years, 16 patients were lost to follow-up (35.6%) and 22 were survivors (48.9%). In addition, the evolution was fatal for 7 patients, a mortality of 15.6%. The death was secondary to breast cancer in five patients, following a myocardial infarction in one patient, and a stroke in the last. Mean overall survival was 142.5 months. Overall survival at 5 and 10 years was 83.2% and 76.8% respectively. Mean recurrence-free survival was 124.9 months. Recurrence-free survival at 5 and 10 years was 64.5% and 58.6%, respectively. Our study showed a correlation between the tumor size and the presence of lymphadenopathy (p = 0.012) as well as the presence of distant metastases (p < 10^-3^) (Table 3).

**Table 1:** clinical characteristics of patients

Nodule		41 (91.1%)
Average size (cm)		3.4 [1.9 - 4.9]
Appearance of the skin		
	Normal	18 (40%)
	Pathological	27 (60%)
Focus on the deep plane		
	Yes	9 (20%)
	No	36 (80%)
Stade T		
	T1	4 (8.9%)
	T2	16 (35.6%)
	T3	2 (4.4%)
	T4	23 (51.1%)
Stade N		
	N0	22 (48.9%)
	N1	16 (35.6%)
	N2	7 (15.6%)
Stade M		
	M0	31 (68.9%)
	M1	7 (15.6%)
	Mx	7 (15.6%)

**Table 2:** histological and immunohistochemical characteristics

Mean histological size (cm)		3
Histological types		
	Invasive ductal carcinoma	43 (95.6%)
	Mucinous carcinoma	1 (2.2%)
	Pure ductal carcinoma	1 (2.2%)
SBR grade		
	I	3 (6.8%)
	II	30 (68.2%)
	III	11 (25%)
Paget's disease of the nipple		2 (5.3%)
Invasive ganglion		25 (67.5%)
Positive estrogen receptors		29 (64.4%)
Positive progesterone receptors		29 (64.4%)
Her2neu		6 (13.3%)
Immunohistochemical types (22 cases)		
	Luminal A	15 (68.2%)
	Luminal B	6 (27.3%)
	Triple negative	1 (4.6%)

**Table 3:** determinants of overall survival rate (OSR)

Factors	Overall survival Rate (%)		P
	At 5 years	At 10 years	
**Age (years)**			
< 60	94.7	82.9	0.001
60-80	83.3	83.3	
> 80	0	-	
**Stade T**			
T1 ou T2	90	90	0.023
T3 ou T4	77.5	58.1	
**Stade M**			
M0 ou Mx	86.4	79.2	0.006
M1	71.4	71.4	
**Histological size (cm)**			
pT ≤ 2	100	87.5	0.040
2 < pT ≤ 5	79.8	79.8	
pT > 5	0	-	
**Occurrence of recurrence**			
yes	67.5	-	0.023
No	96	96	

## Discussion

In men, breast cancer is so rare that it represents less than 1% of all male malignancies and less than 1% of breast cancers [1, 3, 4]. In Tunisia, male breast cancer accounted for 1 to 2.1% of all breast cancers and 0.4 to 1% of cancers affecting humans [2]. The age of onset of the disease varied in the literature between 60 and 70 years with an average age of 67 years [1, 5, 6], shifted from 5 to 10 years or more by comparison to that of the woman. A family history of breast cancer is considered to be one of the most important risk factors [7, 8], with 15 to 20% of men with breast cancer having a family history of breast cancer [9]. Family cases of male cancer are similar to breast cancer in terms of amplitude and earliness. In addition, breast cancer in both parents increases risk in offspring [10]. The prevalence of small, deleterious mutations in BRCA1 and BRCA2 genes in men with breast cancer is well established. Overall, about 15% of all breast cancer cases in men are associated with mutations in the BRCA2 gene [4]. Other genetic factors may be involved in the genesis of male breast cancers such as P53 gene mutation, Klinfelter syndrome but their role is not well documented in the literature [11, 12].

Many studies report a delay between onset of symptoms and timing of diagnosis. This average delay is 14 months in a Moroccan series [6], 18 months in an Algerian series [13] and only 7 months in a Tunisian series [14]. In general, it varies between 6 and 35 months [7]. It can be assumed that men consult late, out of ignorance, ignorance or denial of the disease. For them this pathology is strictly feminine, moreover the practitioners can not pay attention to it because of its extreme rarity. A painless retro-areolar mass is the presenting symptom in 50-95% of men with breast cancer. Often the mammary nodule is found on self-examination (91.5%). Other clinical signs are observed isolated or associated with the breast mass such as: mastodynia, breast discharge (1 to 12%), inflammatory signs, ulceration (4 to 17%), gynecomastia or symptoms related to a metastatic disease (3 to 12%): bone pain, functional impotence, pleuropulmonary signs [14]. Nipple abnormalities are early and more frequent than in women, they are seen in 40 to 50%, perhaps because of the low volume of breast tissue in men and the central location of most tumors [15, 16]. Paget's disease complicates 1.45% of male breast cancers / 0.68% in women. Axillary lymph nodes clinically involved may occur in 40-55% of patients [17, 18].

Because of its limited volume, the male breast is not easy to examine with mammography. Nevertheless, many still consider it the method of choice to complete the clinical examination of this organ. In mammography, male breast cancer usually occurs as a round, oval or irregular high density subareal mass with circumscribed, indistinct, speculative or micro-lobulated margins. The presence of secondary features such as thickening of the skin, nipple retraction and axillary lymphadenopathy facilitate diagnosis [19, 20]. Microcalcifications are rare in breast cancer in men and are observed in only 13% to 30% of cases [20]. Breast ultrasound is very effective in exploring isolated lesions and has become a natural adjunct to diagnosis [20]. The majority of men (84%) presented with a palpable mass, which should be able to be histologically minimally invasive by microbiopsy or fine needle aspiration. Despite the existence of minimally invasive techniques, surgical biopsies continue to be a common practice in men. They are carried out in 35% of cases according to the series [15].

Historically, male breast cancer is often diagnosed at an advanced stage: 81.5% of cases are diagnosed at tumor stages = II and 56.8% have lymph node involvement at the time of diagnosis [21]. A comparative analysis based on data from the National Institute of Surveillance, Epidemiology, and End Results (SEER) for breast cancers recorded between 1973 and 1998 [1] and the period between 2010 and 2012 [16] showed that the frequency of early forms in the initial diagnosis increased over these years for both men and women, but this increase is much more marked for women, and men were more likely to women at very advanced stages of the disease (stage III and IV) (24.9% against 17.2%) (Table 4). Non-specific invasive ductal carcinoma is the predominant histologic type of primary male breast cancer, accounting for approximately 85% of cases by series. Papillary carcinoma is the second most common histological subtype of breast cancer in men, accounting for 2.6% of cases. It is twice more likely to occur in men than in women [20]. Lobular carcinoma of the breast is extremely rare in humans (1 to 1.5% of cases). Invasive mucinous carcinoma accounts for only 1% of male breast carcinomas [19]. In large series performed on male breast cancer, histological grade II was the predominant grade with a frequency of 54 to 58% followed by grade III (17 to 33%) then grade I (12 to 20%) [22]. Axillary lymph node involvement is significantly correlated with histologic tumor size (pT) and SBR grade. These same findings have been proven by our study. Axillary lymph node involvement is more common in men than in women: 30 to 53% in men versus 20 to 45% in women [1]. In our series, the rate of ganglion invasion is 67.5%. Breast cancer in men more frequently expresses hormonal receptors than in women. Estrogen receptors (ER) are positive in 91 to 95% of male cases against 76 to 78%. Progesterone receptors (PRs) are 80% positive in men compared to 67% in women [23]. Other hormone receptors may be expressed by mammary neoplastic cells such as androgen receptors (AR), which are seen in 34-95% of cases of male breast cancer [24]. Sixteen percent (16%) of breast cancers in men over-express her and this using both immunohistochemical analysis (3+) and the FISH technique [25]. The median Ki67 values in men were consistent with those found in women, indicating that there are no major differences in the prevalence of expression of proliferation markers between breast cancers in the two sexes [26]. Mastectomy associated with ipsilateral axillary dissection remains the standard surgical treatment in men [27]. Even for SDC, total mastectomy is the preferred surgical option in men, resulting in a very low recurrence rate [28]. The possibility of conservative breast surgery and radiotherapy is poorly supported in the literature [28].

**Table 4:** comparative analysis of breast cancer data recorded between 1973-1998 and 2010-2012 between men and women

STADE	Men		Women	
	[1] : N = 2537	[30]: n = 1442	[1]: n = 383 146	[30]: n = 172 847
I	29.4%	36.3%	38.4%	50.9%
II	38.5%	38.8%	27.7%	31.9%
III	7.5%	17.6%	5.3%	11.4%
IV	5.7%	7.3%	3.9%	5.8%

Axillary dissection reduces the risk of axillary recurrence by excision of metastatic lymph nodes. Indeed, and according to a meta-analysis on male breast cancer, axillary lymph node dissection reduces the regional lymph node recurrence rate to 1.2% compared to 13% in the absence of axillary dissection [29]. The sentinel lymph node technique has been extensively studied in patients with early-stage breast cancer. But given the scarcity of male breast cancer, it is unlikely that this technique will be evaluated as rigorously in men. Adjuvant locoregional radiotherapy was administered more frequently to men with breast cancer than to women because the disease was more advanced locally in men and considered more aggressive [30]. The role of adjuvant chemotherapy is less well established in male breast cancers; however, chemotherapy is generally considered for a medium to high risk disease, particularly a hormone-negative disease, or for patients who have become refractory to hormone therapy [29,31]. Chemotherapy is also recommended as an initial treatment for breast cancer patients with rapidly progressive or life-threatening visceral diseaseas [29]. Medical hormonal treatments include anti-androgens, estrogens, progestins, aromatase inhibitors and tamoxifen. These options are generally more preferable to men than orchiectomy. As most breast cancers in men are estrogen-receptor positive, tamoxifen is generally the standard adjuvant therapy and is also recommended as an initial treatment for metastatic hormone receptor-positive breast cancer [29, 31, 32]. Another hormone therapy that has been developed is anti-androgens. Anti-androgen therapies may be an alternative and complementary treatment option for patients with triple-negative breast cancer with limited therapeutic possibilities [33]. The comparison of overall prognosis for male and female breast cancer patients is controversial. Studies reporting a poor prognosis for men have suggested that male breast anatomy may provide fewer barriers to metastasis or that more aggressive tumor biology may be the basis for survival variation [16]. However, others have found that, once separated by lymph node stage or involvement, the prognosis is the same. The reported 5-year overall survival rate for male breast carcinoma varies considerably from 40% to 80% with a specific survival rate of 89% and 72% at 5 years and 10 years respectively [29].

## Conclusion

Breast cancer in men presents only 0.8% of breast cancers in the Tunisian center confirming the rarity of this pathology. It occurs at a later age compared to women. It is often diagnosed at an advanced stage with a high rate of lymph node involvement and distant metastases. This study allowed us to show that breast cancer in men has certain peculiarities compared to that of women. However, its management is still dictated by the studies carried out on the female population.

### What is known about this topic

- Breast cancer in males is relatively rare and Immunohistochemical profiling studies carried out on female breast cancer has been extrapolated to breast cancer in males.

### What this study adds

- This study allowed us to show that breast cancer in men has certain peculiarities compared to that of women.
